# Supplementary material for: Are carbon emissions trading and green financial instruments synergistic? -Comprehensive quantitative research based on content analysis
Source: PLoS One. 2024 Mar 7;19(3):e0298601. doi: 10.1371/journal.pone.0298601 (PMC10919601; doi:10.1371/journal.pone.0298601)
Supplement: S1 Data — (ZIP) [file pone.0298601.s001.zip › Data/地级市Z_F工作报告.zip!/╔╜╢1⁄2/┴─│╟╩╨/└┤╫╘╣1⁄2╓┌║┼í╛┬φ┐╦╩2╛▌═°í┐_2015.docx]

各位代表：

现在，我代表市人民政府向大会报告工作，请予审议，并请市政协各位委员提出意见。

一、2014年工作回顾

刚刚过去的 2014年，是令人难忘、很不平凡的一年。一年来，面对复杂严峻的国内外环境和艰巨繁重的发展任务，全市上下在省委、省政府和市委的坚强领导下，紧紧围绕“东融西借、跨越赶超，建设冀鲁豫三省交界科学发展先行区”的奋斗目标，团结拼搏，真抓实干，经济社会保持了良好的发展势头，市十六届人大四次会议确定的主要目标任务圆满完成。

（一）综合实力明显提升。在宏观经济下行压力较大的形势下，全市经济“逆势上扬、进中向好”。生产总值完成2516亿元，增长9.4%，高于全省0.7个百分点。地方一般公共预算收入完成156.2亿元，增长15.2%，高于全省5个百分点，增幅居全省第2位。国税、地税收入均突破百亿元，其中国税收入完成105亿元，入地方库部分增长22.2%，增幅居全省第2位；地税收入完成103亿元，增长16.9%。地方一般公共预算收入占生产总值比重达到6.2%，税收占地方一般公共预算收入比重达到73.6%，实现了速度质量双提升。全市财政总收入突破400亿元，达到446.6亿元。

（二） 结构调整明显加快。全市三次产业比例调整为12.4∶51.5∶36.1。现代农业加快推进。粮食总产完成123亿斤，首次达到“吨粮市”标准，被评为全国粮食生产先进单位。瓜菜菌总产达到1602万吨，保持全省第一，被中国蔬菜流通协会评为“中国蔬菜第一市”。农产品质量可追溯体系在全省唯一实现全覆盖，新增绿色食品397个,居全国地级市第1位。全市高标准农田面积达到410万亩，农业机械化率达到86%，我市以全省第1名的成绩被评为国家现代农业示范区。工业经济加快升级。规模以上工业企业达到2602家，增加115家；主营业务收入达到8593亿元，增长14.6%，增幅居全省第4位。科技投入与创新力度加大，全市完成投资过5000万元技改项目321个，技改投入达到481亿元，增长21.2%。信发集团成功创建国家资源综合利用“双百工程”骨干企业，中色奥博特被评为国家技术创新示范企业，祥光铜业在全省唯一荣获中国专利奖金奖，蓝山集团荣获国家技术发明二等奖，东阿阿胶荣获省长质量奖个人奖。全市高新技术产业产值达到2145亿元，占规模以上工业比重达到24.9%，提高了2个百分点，增幅居全省第1位。现代服务业加快发展。龙头景区景点加快建设，中华水上古城正式开街运营，阿胶养生文化苑基本建成，我市被国土资源部授予“中国温泉之城”。电子商务快速兴起，电商企业达到3440 家。全社会消费品零售总额达到924.7亿元，增长12.7%，增幅居全省第4位。战略新兴产业加快崛起。中通新能源客车产量增长110%，增幅居全国同行业第1位，时风电动汽车产能达到20万辆，莘县巨龙电动车正式投产，聊城已经成为全国新能源汽车生产基地；建成生物制药研发平台9个，投产新产品11个，聊城正在成为全省重要的生物制药研发生产基地。各位代表，过去的一年，全市上下坚持把转方式调结构作为主攻方向，经济发展不仅速度快、效益好，而且产业结构、产业层次都有了明显提升，全市经济转型升级取得了实质性进展、迈出了坚实的步伐！

（三）发展后劲明显增强。全市固定资产投资完成1833亿元，增长16%，增幅居全省第4位。招商引资取得新成绩。全市到位资金851.7亿元，增长15.6%。在省委、省政府举办的全国知名民营企业入鲁助推转调创活动中，我市共签订招商项目合同 232个，签约额达到2117亿元，居全省第3位，汉能控股、英利集团等一批知名企业落户聊城。项目建设取得新成效。全市在建过亿元项目589个，增加149个，其中过10亿元项目86个，过百亿元项目10个，完成投资958亿元，增长17.7%。重大交通项目取得新进展。邯济铁路复线、济聊一级公路聊城段建成通车，德商高速加快建设，青兰高速、莘县至南乐高速已经开工。郑济客专、聊泰铁路、京九高铁、聊黄铁路、聊石铁路、德郓高速、军民合用机场、徒骇河通航等，全部列入国家和省综合交通网发展规划。各位代表，通过去年一年的努力，全市人民共同期盼的水陆空立体大交通体系已经初具基础，高速成网、高铁叠加、飞机起降、轮船出海的宏伟蓝图正在一步步变为现实！

（四）发展活力明显提高。“一区一圈一带”战略红利加速释放，我市获批享受中部地区产业政策，各项改革全面推进，经济社会发展呈现新的生机和活力。市场主体大幅增加。全年累计登记各类市场主体24.4万户，增长34.8%，增幅居全省第3位。农业经营体制创新加快。全市60.9%的村完成土地承包经营权确权颁证，新增土地流转面积50.4万亩；农民合作社达到9084家，增加1578家，实现了“一村一社”全覆盖。金融改革发展成效显著。金融机构各项存款余额达到2289亿元，增长18%；贷款余额1700亿元，增长19%，增幅分别居全省第1位和第4位。10家企业在“新三板”挂牌上市，居全省第3位；境外上市取得新突破，高唐天泰集团成功在英国创业板上市。县域经济增势强劲。7个县（市、区）和经济技术开发区地方一般公共预算收入突破10亿元，茌平县在全国百强县中由81位上升到75位，所有省级经济开发区在全省综合考核中均提高了3个位次以上。

（五）城乡面貌明显变化。全市城镇化率提高1.9个百分点，居全省第2位。城市规模进一步扩张。“五环聊城”四环道路建设基本完工，中心城区主框架达到230平方公里。各县（市）城区规模明显扩大，新增建成区面积50平方公里。城市改造进一步加快。96个项目、3.9万户列入全省棚改计划，获得中央和省补助资金17亿元，均居全省第1位；开工建设回迁安置项目195个、9.7万套，其中基本建成6.8万套，4.3万户群众乔迁新居。城市功能进一步完善。全市新建改建城乡道路1780公里，新增绿化面积2047公顷，新增供热管网110公里，新增雨污分流管网689公里，均相当于过去10年的总和。市民文化活动中心投入使用，体育公园被评为国家Ⅰ类地震应急避难场所。中国智慧城市研究院在聊城成立，我市成为国家智慧城市试点市。农村环境进一步改变。“户集、村收、镇运、县处理”的环卫体系基本形成，在全省群众满意度测评中名列第2位。各位代表，去年全市住房和城乡建设总投资突破1000亿元，城乡变化进一步加大，城区路更宽更平、绿更多更美、灯更炫更亮，农村更干净更整洁，我们的家园更加宜居、更加美丽了！

（六）群众生活明显改善。全市财政用于民生的支出达到197亿元，占财政总支出的68.9%，去年提出的“十件民生实事”全部落实。全市城镇居民人均可支配收入达到28382元，农民人均纯收入达到11232元，分别增长8.8%和11.4%。生态环境更加优美。全市林木绿化率达到37.5%，蓝天白云天数比上年增加50%，是全省空气质量综合改善幅度最大、获得生态补偿最多的市。在全省唯一入选国家节能减排财政政策综合示范城市，3年内将争取国家、省补助资金57亿元。就业和社会保障更加完善。在全省率先建成了创业大学，设立了5000万元的市级创业带动就业扶持资金，城镇新增就业6.4万人，农村劳动力转移就业8.6万人，城镇登记失业率控制在2.94%。提高了七大类社会保障标准，大病保险最高补助额达到20万元，城市低保、农村低保、五保集中供养标准每人每年分别提高了360元、200元和1200元。全市新建农村幸福院126处，新增社会养老床位6305张。社会事业更加进步。教育教学条件明显改善，135个乡镇公办中心幼儿园全部投入使用，可容纳900名幼儿的市实验幼儿园开工建设；新建改建农村校舍135处；中心城区新建扩建中小学11处，新增教学班152个，班额过大问题得到有效缓解。文化事业更加繁荣，三段运河、6个遗产点列入世界文化遗产名录，文化惠民“三千工程”超额完成。卫生计生事业健康发展，市人民医院以第1名的成绩率先通过省级区域医疗中心验收，市中医院在全省率先成立了中医医疗联合体。中心城区新增新能源公交车200辆，群众出行条件明显改善。社会更加和谐稳定。法治聊城建设和平安创建活动深入开展，群众安全感和满意度明显提升；全市没有发生重特大安全生产事故，事故起数、死亡人数、受伤人数和直接经济损失全面下降。双拥优抚安置政策全面落实，慈善接收捐款增长34%；审计、民族、社科等工作荣获国家级荣誉称号，广播电视、妇女儿童、外事侨务、残联、人防、气象等工作荣获省级荣誉称号，新闻出版、档案史志等工作取得新的成绩。

（七）工作作风明显转变。各级深入开展党的群众路线教育实践活动，“四风”问题得到有力整治。全市调整清理多占办公用房4.3万平方米，清理清退超标公务车108辆；市政府发文减少了30%，会议数量减少了10%，“三公”经费降低了20.7%。政府服务水平明显提高，建设了网络信息办公系统，在全省率先实行了“四证联办”、“先照后证”。积极推进政务公开，对市长公开电话进行了升级改造，接通率、办结率均达到99%以上，居全省第1位，被群众称为“24小时不下班的政府”。严格执行人大及其常委会的决定、决议，加强与政协民主协商，人大代表建议和政协提案办理满意度均达到98%以上。严格落实党风廉政建设责任制，查处违反中央“八项规定”及“庸懒散”等问题430起，处理579人，维护了政府良好形象。

各位代表，一年来，通过各级各方面的共同努力，全市经济社会发展全面突破、成效明显。一年来的实践充分表明，全市上下干事创业的氛围更浓。围绕建设冀鲁豫三省交界科学发展先行区的奋斗目标，全市都在想发展、议发展、谋发展、促发展，思想高度统一，工作坚强有力，注意力、兴奋点都始终聚焦在发展上。敢为人先的胆识更高。坚持高起点、高标准，逢一必争、逢旗必夺，敢与高的比、敢与快的赛、敢与强者竞高低，全市有17项工作排在全省前3位，有的排在全国前列。攻坚克难的闯劲更足。在大气污染防治、回迁安置房建设、城乡环卫一体化、化解金融风险等方面，敢于担当，勇于负责，破解了难题，打开了局面。勤政为民的作风更实。广大党员干部工作作风切实改进，落实能力明显增强，工作效率全面提升，贯彻党的群众路线、持续改进作风的长效机制基本形成。团结拼搏的合力更强。各级领导带头实干、以上率下，各级干部爱岗敬业、尽职尽责，各位企业家奋力拼搏、二次创业，广大群众兢兢业业、踏实苦干，全市上下一盘棋、一条心，凝聚力和战斗力进一步增强。各位代表，全市共同营造的这种生动局面，是聊城最大的优势、最强的底气。只要我们巩固好、发展好这种良好局面，聊城的各项事业将无往而不胜，跨越赶超的目标就一定能够实现！

各位代表，去年的成绩来之不易。这是省委、省政府和市委正确领导的结果，是市人大、市政协和社会各界大力支持的结果，是全市上下共同拼搏的结果。在此，我代表市人民政府，向全市工人、农民、知识分子和企业家，向各民主党派、工商联、各人民团体和各界人士，向驻聊人民解放军、武警官兵、公安干警和中央、省驻聊各单位，向所有关心、支持聊城发展的海内外朋友，表示衷心的感谢！

各位代表，在看到成绩的同时，我们也要清醒地认识到，我市在发展中还存在不少问题和不足。全市综合实力依然不强，人均占比仍然较低；产业结构依然不够合理，服务业占比低，重化工业比重大；科技创新能力依然薄弱，高新技术产业占比还比较低；经济外向度依然不高，对外开放力度还不够大；节能减排压力依然较大，大气污染防治任务仍很艰巨；社会治理和民生工作依然存在薄弱环节，有些群众生活还比较困难；政府职能转变依然不到位，营商环境有待进一步优化。对这些问题，我们一定要高度重视，下决心予以解决。

二、2015年的目标任务

各位代表，2015年是全面深化改革的关键之年，是全面推进依法治国的开局之年，是全面完成“十二五”规划的收官之年，也是我市打好科学发展、跨越赶超三年攻坚战的决战之年。做好今年的政府工作，必须把思想统一到中央关于我国经济发展进入新常态的重大战略判断上来，主动认识新常态、适应新常态、引领新常态，做到“调速不减势、量增质更优”，努力开创全市经济社会发展的新局面。

今年政府工作的总体要求是：全面贯彻党的十八大、十八届三中、四中全会和习近平总书记系列重要讲话精神，坚持稳中求进工作总基调，坚持以提高经济发展质量和效益为中心，适应新常态，抢抓新机遇，展现新作为，着力在转方式调结构、实施创新驱动上有新突破，在深化改革扩大开放、激发市场活力上有新成效，在加强社会治理、保障和改善民生上有新进展，在推进依法行政、加强政府自身建设上有新提升，全力打好跨越赶超攻坚战第三战役，为建设冀鲁豫三省交界科学发展先行区奠定坚实基础。主要预期目标是：生产总值增长10%左右，地方一般公共预算收入增长11%左右，固定资产投资增长16%左右，社会消费品零售总额增长12.5%左右，实际利用外资增长10%左右，进出口总额增长6%左右，城镇居民人均可支配收入增长10%左右，农民人均纯收入增长11%左右，居民消费价格涨幅控制在3%以内，城镇登记失业率、节能减排、人口自然增长率等全面完成省下达的目标任务。

工作中，重点抓好以下九个方面：

（一）以更大力度推动经济稳定增长，确保在综合实力提升上实现突破。坚持把发展作为聊城的最强音，确保经济增长势头更加强劲。一是增强区域发展战略的带动力。紧紧抓住“一区一圈一带”重大机遇，加大规划项目推进力度，抓好“聊茌东大三角”建设，积极争取享受中部地区财政政策和革命老区政策。广泛参与“一区一圈一带”和环渤海、京津冀等区域合作，促进优势互补、共同发展。二是增强优质投入的拉动力。实施重点项目“六百工程”，围绕工业、农业、服务业、城建、民生、招商引资等六大领域，各实施重点项目100个，600个项目总投资7300亿元，年内完成投资2300亿元，其中过10亿元项目171个，过100亿元项目20个。强化资金、土地等要素保障，挖潜改造土地1.1万亩，增减挂完成验收1万亩。三是增强民营经济的内生力。实施创业创新工程，加大对大众创业的财税支持和金融服务，确保新增个体工商户和私营企业5万户，新增规模以上中小企业200家，民营经济税收增长20%以上。四是增强重大交通设施的支撑力。加快青兰高速、莘县至南乐高速建设，确保德商高速建成通车；争取军民合用机场开工建设，郑济客专、京九高铁、聊泰铁路、聊黄铁路、聊石铁路、德郓高速、徒骇河通航等取得扎实进展。抓好干线公路建设，新建改建农村公路500公里。各位代表，面对经济发展新常态，我们必须咬住发展不放松，在确保质量效益的前提下，积极进取、挖掘潜力，力争发展得更快一些，坚定不移地实现跨越赶超的奋斗目标！

（二）以更大力度推进转方式调结构，确保在经济提质增效上实现突破。坚持在发展中转型、在转型中发展，着力推动传统产业向中高端迈进，切实增强战略新兴产业和服务业的支撑作用。

大力发展精致农业。紧紧抓住列入国家现代农业示范区的机遇，深入实施“农业龙头带动”战略，努力打造北方平原地区精致农业先行区。一是推进农业生产设施化。扎实开展农田水利基本建设三年会战，抓好小农水重点县、中小河流治理、南水北调平原水库建设，治理沟渠599条、1200公里。落实农机补贴资金1.4亿元，建设粮食高产创建示范田100万亩，粮食总产稳定在120亿斤以上。二是推进农业产业高端化。以开展“抓带增”活动为抓手，加大龙头企业培植力度，抓好凤祥集团千亿食品产业园建设，新增省级以上龙头企业10家，规模以上龙头企业产值突破1100亿元；集中力量培植莘县蔬菜、丁马甲鱼等17个区域公共品牌，抓好百万头肉驴养殖等100处高效生态农业示范基地建设。三是推进农业管理精细化。大力推进精耕细作，鼓励采用智能温室、物联网等先进技术，发展生态循环农业。完善监管、检测、执法和质量安全追溯体系，新增“三品一标”80个，打造全国最大的绿色安全蔬菜生产基地。加快农产品市场体系建设，支持发展直销、配送、电子商务等物流业态。四是推进农业科技集成化。围绕现代育种、生物农业等技术集成领域，抓好5个生物农业科技孵化器、10个区域研发平台、100个新品种新技术展示基地建设，打造我国北方最大的优质产品良种研发与供应中心。推行新型职业农民培训管理与认证，年培训20万人次，用三年时间把核心经营主体培训一遍。

推进工业转型升级。大力实施“万亿工业”战略，更加注重发挥企业家才能，更加注重科技进步和全面创新，确保规模以上工业主营业务收入突破1万亿元。加快传统产业改造，实施八大传统产业转型升级工程，推进信息技术与传统制造业跨界融合，抓好汉能光伏、诺伯特机器人等145个项目建设，基本完成鲁西集团三个分厂退城进园项目，确保技改投入增长17%以上。加快骨干企业膨胀，实施“1150”工程，力争主营业务收入过千亿元的企业达到1家，过百亿元的达到10家，过50亿元的达到10家。加快特色产业集聚，抓好“3+5”千亿产业园建设，力争主营业务收入达到3600亿元。加快新兴产业培育，重点培植新能源汽车、生物制药、先进装备制造、节能环保等四大产业，抓好中通新能源客车、山东抗体制药协同创新中心等48个项目建设，确保高新技术产业产值比重提高2个百分点以上。

发展壮大服务业。实施服务业倍增战略，努力做大规模、提升水平，确保服务业增加值占比提高2个百分点。一是大力发展商贸物流业。围绕打造现代仓储和物流集散基地，抓好聊城物流园区等10大园区建设，物流业增加值增长15%以上。二是加快发展文化旅游业。把旅游业作为传统服务业转型升级的突破口，围绕打造中国首选养生旅游目的地城市，抓好江北水城旅游度假区、黄河天音风情区、马颊林海度假区、东昌古郡游憩区等精品景区建设。加快中华水上古城建设步伐，强化文化内涵，提升知名度和影响力。积极发展乡村旅游，重点建设10个旅游特色小镇、40个旅游特色村。建设聊城旅游集散中心，打造水浒旅游、水文化旅游等一批精品线路。三是积极发展新兴服务业。以创建国家电子商务示范城市为目标，抓好聊城电子商务产业园等10个电子商务示范园区、鑫鹏E钢在线等100个电子商务重点项目建设，确保电子商务交易额增长30%以上，力争突破1000亿元。加快发展金融保险、研发设计、信息技术、检验检测等生产性服务业，抓好“3+5”千亿产业园服务业集聚区、金融服务一条街和聊城国家级质检中心建设，推进服务业与一二产业协同发展、跨界发展。积极培育健康养老、家政服务、教育文化、体育健身等生活性服务业，努力满足人们多层次、多元化消费需求。各位代表，面对经济发展新常态，我们既要大力提升传统产业，更要善于发现和培育新产品、新业态，努力让老树开出新花，让新芽长成大树，为聊城跨越赶超提供强有力的支撑！

（三）以更大力度实施创新驱动，确保在科技人才支撑上实现突破。大力实施创新驱动战略，加快建立企业为主体、市场为导向、产学研相结合的技术创新体系。一是加强企业创新。实施企业创新能力提升工程，加大对企业创新发展的扶持力度，力争培育省级以上技术中心10家，认定市级企业技术中心25家。二是加强平台建设。深化与聊城大学等科研院所的合作，共建创新平台，共筑人才高地。加快鲁西大学科技城、高新技术企业孵化中心建设，尽快构建起开放共享的创新平台体系。三是加强人才培养引进。实施重大人才工程，加大高端人才和高技能人才引进培养力度。支持高等教育、职业教育和民办教育发展，构建鲁西技能型人才培养高地。鼓励企业健全股权、期权分红等激励机制，切实做到吸引人才、留住人才、人尽其才。各位代表，实现聊城跨越赶超，希望在创新，关键在人才。我们要在全社会进一步营造激励创新、重视人才的良好环境，让一切想创新的人有机会、能创新的人有舞台，让创新之树根深叶茂、四季长青！

（四）以更大力度全面深化改革，确保在发展活力增强上实现突破。坚持把改革作为转方式调结构、实现跨越赶超的制胜法宝，全面推进各领域改革。一是推进农村改革。基本完成农村土地承包经营权确权颁证工作，促进农业适度规模经营。大力培育新型经营主体，积极开展新型农村合作金融试点，扩大农村集体产权制度改革试点范围，全面建成农村产权综合交易平台。二是推进企业改革。深化国有资本运营和监管体制改革，完善公司法人治理结构，鼓励发展混合所有制经济。引导规模以上企业进行公司制改造，推动民营企业建立现代企业制度。三是推进财税金融改革。研究设立政府引导基金和专项私募基金，采取政府购买服务、政府与社会资本合作等模式，提高财政资金使用效率。加快培育大型融资担保机构，积极帮助中小企业解决贷款难、贷款贵问题。大力推进企业走向资本市场，力争新增“新三板”挂牌上市企业10家、股权挂牌企业10家。四是推进行政管理体制改革。全面完成市、县、乡政府机构改革任务，按照“接、放、管”的要求，理顺市县两级行政审批体制，进一步激发县域经济发展的活力。五是推进城乡发展一体化改革。深化户籍制度改革，促进城镇常住人口市民化。抓好农村新型社区建设试点，有序推进“村改居”。各位代表，面对新常态，展现新作为，我们必须向改革要动力，最大限度地激发市场和社会活力，努力使改革新红利转化为发展新动力！

（五）以更大力度扩大对外开放，确保在外向型经济发展上实现突破。紧紧抓住国家实施新一轮高水平对外开放的机遇，积极融入“一带一路”战略，进一步提高对外开放水平。一是坚定不移地抓好招商引资。继续加大招商引资力度，以100个重点招商推进项目为抓手，确保到位资金突破1000亿元，力争每个县（市、区）至少引进1个过千万美元的外资项目。着力提高招商引资的质量，努力在招大引强上实现突破。着力增强招商活动的针对性、实效性，深化与各地商会和中介机构的合作，充分发挥企业的主体作用，更多地开展委托招商、产业招商、以商招商。二是努力促进外贸稳定增长。立足我市优势产业，积极培育新的外贸增长点，力争出口增长20%。抓住国际市场大宗商品价格走低的时机，扩大资源性产品、关键设备及零部件进口。加强海关特殊监管区建设，重点抓好开发区保税物流中心、冠县宝信物流园区、临清保税园区建设，开通大进大出的便捷通道。三是安全高效建设“海外聊城”。支持骨干企业走出去，建立境外资源和生产基地，重点抓好泉林集团美国制浆造纸、信发集团牙买加氧化铝等项目建设。四是积极推动开发区转型升级。进一步创新管理体制机制，着力培植特色产业，提升科技含量、投入产出、质量安全等标准，确保所有省级经济开发区在全省综合排名位次前移。

（六）以更大力度加快新型城镇化，确保在“双百大城市”建设上实现突破。以建设“宜居宜业宜游”城市为目标，着力在提升层次、保障质量、精细管理上下功夫，力争城镇化率提高3个百分点。坚持高起点规划。完成聊城市总体规划编制，加快县城、乡镇总体规划和村庄规划编制，形成覆盖全域的城乡规划体系。抓好专项规划、控制性详细规划和重点项目规划编制，确保在科学布局、彰显特色上实现新提升。坚持高水平建设。突出抓好回迁安置房建设，严把时间关、质量关和配套关，建设回迁安置项目60个，确保完成3.5万套，让群众早日搬入新居。完善城镇路网体系，全市新建改建城镇道路300公里，中心城区全面完成内外四环道路建设，打通断头路，完善“微循环”。加强地下管线建设，推进雨污分流和各类管线入地，提高城市防洪排涝能力。实施小城镇改貌工程，按照小城市标准加强省市级示范镇建设。抓好30个农村新型社区建设，社区规模达到3000人以上。坚持高效能管理。按照“平明绿美净齐”的要求，进一步推进城市精细化管理，加强对违规经营、乱停乱放、乱贴乱画的治理，确保市容市貌、交通秩序明显改善。全面推进生态文明乡村建设，巩固提升城乡环卫一体化成果，深入开展乡村文明行动。各位代表，聊城城乡建设的框架已经拉开，我们必须乘势而上，推进城镇建设与生态环境相结合、与文化传承相结合、与产业发展相结合，实现城镇化速度质量双提升，确保“双百大城市”三年攻坚战取得决定性胜利！

（七）以更大力度抓好节能减排，确保在生态环境改善上实现突破。紧紧抓住创建国家节能减排财政政策综合示范城市的机遇，大力实施“六化”工程。一是产业低碳化。严格产业准入，加强对高耗能企业的监管，推进现有电厂“上大压小”。大力推广天然气等清洁能源，建设液化天然气调峰储备中心。深入开展百企节能低碳行动，抓好121个重点节能项目建设，完成投资97亿元。二是交通清洁化。加快国家新能源汽车示范市建设，抓好黄标车淘汰、油品升级和油气回收治理。建设城市慢行系统，新建自行车专用道100公里。三是建筑绿色化。对公共建筑、公益性建筑全面推行绿色建筑标准，旅游度假区严格按照绿色建筑示范区标准建设，完成既有建筑节能改造50万平方米。四是主要污染物减量化。加快推进燃煤企业脱硫、脱硝和除尘升级改造，加强城区扬尘污染防治，新增湿地保护面积3万亩，林木绿化率达到40%，努力争创国家森林城市。五是可再生资源利用规模化。推进垃圾分类处理和资源化利用，抓好信发集团赤泥粉煤灰利用和临清、阳谷、茌平垃圾焚烧发电厂建设，新增太阳能发电150兆瓦,推广太阳能光热建筑一体化80万平方米。六是生态监管法治化。严格依法开展环境监管执法，对环保违法行为零容忍。各位代表，人民群众对清新空气、清澈水质、清洁环境的需求，就是我们努力的方向。我们一定要顺应全市人民的热切期盼，更加扎实地做好工作，努力让聊城的天更蓝、水更清、空气更清新！

（八）以更大力度加强文化建设，确保在文化大发展大繁荣上实现突破。聊城历史悠久，文化灿烂，名人志士辈出，文物古迹众多。我们必须挖掘和利用好丰富的文化资源，着力增强文化软实力，努力把聊城打造成为思想基础巩固、服务体系健全、产业优势突出、发展活力强劲的文化强市。一是抓好文化资源挖掘阐发。深入挖掘聊城优秀的传统文化，打造一批优秀传统文化传播基地。加强对黄河文化的挖掘，建设黄河森林公园，打造百里生态长廊；加强对运河文化的挖掘，抓好山陕会馆、临清钞关，以及张秋、七级等古镇的保护和利用；加强对水浒文化的挖掘，抓好景阳冈旅游区扩建，深化区域合作，共同打造水浒故里文化旅游目的地品牌；加强对红色文化的挖掘，建设好刘邓大军强渡黄河战役纪念园、鲁西第一党支部等重点项目，抓好历史性建筑和革命遗址保护，打造聊城革命老区品牌；加强对民俗文化的挖掘，抓好雕刻葫芦、剪纸、面塑等非物质文化遗产的生产性保护。实施“乡村记忆工程”，延续地方历史文脉。二是抓好优秀文化宣传普及。充分发挥孔繁森纪念馆等纪念场馆的宣传教育作用，大力弘扬优秀文化。开工建设市图书馆新馆和档案馆新馆，完善现代公共文化服务体系。利用聊城历史文化资源优势，加强文化艺术创作，力争推出一批在全省、全国有重大影响的精品力作。举办好反法西斯战争胜利70周年纪念活动，大力弘扬赵伊坪、范筑先、马本斋等革命烈士扬威不屈的民族精神，激发全市人民的爱国热情。三是抓好文化产业培育发展。推进文化与经济融合发展，围绕文化创意、影视制作、演艺娱乐、文化会展、动漫游戏等五大领域，培植25个文化产业示范基地，努力把聊城打造成为全省文化产业跨越发展的隆起带。

（九）以更大力度保障改善民生，确保在促进社会和谐稳定上实现突破。大力实施“民生优先”战略，加快发展各项社会事业，努力让广大群众享受更多发展成果。

积极推动社会治理创新。加强法治聊城建设，深入开展普法宣传教育，推进公共法律服务均等化；实施涉法涉诉信访改革，切实纳入法治轨道。加强社会信用体系建设，全面抓好政务诚信、商务诚信、社会诚信，加快市级公共信用信息平台建设，打造诚信聊城品牌。加强城乡社区规范化建设，着力增强社区功能。加强人口计生工作，提高出生人口素质。健全农村留守儿童、妇女、老年人关爱服务体系，做好关心下一代工作。深入推进平安聊城建设，切实为人民群众安居乐业创造良好的社会治安环境。强化安全生产监管，坚决遏制重特大安全事故发生。高度重视国防动员、双拥共建和民兵预备役工作，认真做好民族宗教、外事侨务、防震减灾、对台、人防等工作，确保社会和谐稳定。

扎扎实实为群众办实事。围绕关系群众切身利益的困难和问题，着力办好“十件民生实事”。一是提升就业水平。加强聊城创业大学建设，完善公共就业服务体系，免费开展职业技能和创业培训5.5万人，新增城镇就业5万人、城镇失业人员再就业1.5万人、农村劳动力转移就业7万人，让更多的群众掌握一技之长，实现充分就业。二是提升社保水平。每人每年农村低保标准提高到2500元，五保集中供养标准提高到5000元，城乡居民基础养老金标准提高到1020元，城乡居民医保财政补助标准提高到380元，基本公共卫生服务经费财政补助标准提高到40元，进一步提高城市低保、企业退休人员养老金、失业保险金等标准，努力让群众生活更有保障。三是提升办学水平。加强农村中小学建设，对148所学校实施校舍标准化建设，对110所学校食堂进行改造提升，确保让学生喝上热水、吃上热饭；中心城区新建改建中小学9所，建成市实验幼儿园，启动市特殊教育学校建设，全面完成中小学旱厕改水厕工程；推进基础教育综合改革，切实减轻学生课业负担。四是提升医疗卫生服务水平。抓好省级区域医疗中心、中澳国际合作医院等项目建设，扎实推进县级公立医院改革，实施农村贫困家庭先心病儿童免费手术等“四大免费工程”，在乡镇卫生院全面建设标准化国医堂，积极推进医疗卫生服务进社区、进家庭活动，努力让群众就医更加便利。五是提升城市宜居水平。开工建设各类保障性安居工程5.2万套，其中公共租赁住房7967套。中心城区新建提升公园3处，新增集中供热面积100万平方米，新建改建公共卫生间100座，对50个老旧小区进行改造提升，努力让市民生活更加舒适。六是提升交通服务水平。中心城区新增新能源公交车200辆，新增优化公交线路13条；新建停车场11处，增加停车位2000个，努力让市民出行更加便捷。七是提升文化体育服务水平。实施“一村一场戏”工程，新建改造综合性文化服务中心1000个。新增城市社区全民健身工程80处、农村730处，90%的城市社区建成15分钟体育健身圈。八是提升养老服务水平。加强社会养老服务体系建设，新增养老床位4000张、城市社区日间照料中心50处、农村幸福院115处，努力让老年人更好地安度晚年。九是提升食品药品安全水平。完善市、县、乡、村四级食品药品监管网络，加大初级农产品监管力度，主要农产品抽检合格率达到98%以上，打造食品药品安全放心城市。十是提升扶贫开发水平。选择100家企业带动200个贫困村，在150个贫困村派驻“第一书记”，帮助10万农民群众脱贫奔康。各位代表，谋民生之利，解群众之忧，是政府义不容辞的职责。在认真办好“十件民生实事”的基础上，要继续倾听广大群众的诉求和呼声，竭心尽力为全市人民解难题、办实事，努力让全市人民生活得更安心、更放心、更舒心！

三、加强政府自身建设

主动适应新常态、打好三年攻坚战，对政府自身建设提出了新的更高要求。各级政府必须坚持从严治政、改革创新，在建设高效、责任、务实、法治、廉洁政府上取得新的更大进展。

（一）加强高效政府建设，行政效能要有新提升。推进科学发展、跨越赶超，必须提升行政效能。要加快推进政府职能转变，严格执行行政审批目录清单，尽快出台行政权力清单、政府责任清单、市场准入负面清单，向社会公开并接受监督。加强政务服务中心建设，推行网上审批、联合审批，杜绝多头办理、体外循环。进一步清理规范中介机构，实现与挂靠行政单位彻底脱钩，引入竞争机制，提升服务水平。全面优化政府工作流程，进一步加强电子政务建设，努力提升公共服务效能，让群众办事更加省心、省时、省钱。

（二）加强责任政府建设，群众观念要有新增强。作风建设永远在路上。要进一步巩固和拓展党的群众路线教育实践活动成果，驰而不息纠正“四风”，切实把为民作为工作的原点。坚持领导干部联系群众制度，经常深入基层，了解群众疾苦，倾听群众呼声。做好群众来信来访、市长公开电话工作，切实维护群众利益，认真解决群众反映的困难和问题。坚持问题导向，办好民生实事，全心全意为群众搞好服务，切实做到守土有责、守土尽责。

（三）加强务实政府建设，工作作风要有新改进。为政贵在行，关键在落实。要进一步改进工作作风，以奋发有为的精神状态狠抓工作落实，切实做到议而有决、决而有办、办而有责、责而有究。加强政府绩效管理，搞好任务分解，一级抓一级、层层抓落实。深化“庸懒散”专项治理，坚决纠正懒政、怠政、为官不为等现象。加大督查工作力度，扎实抓好全面督查、专项督查，引入社会力量搞好第三方评估，确保各项工作落实到位、富有成效。

（四）加强法治政府建设，依法行政要有新举措。法律的生命力在于实施，法律的权威也在于实施。行政机关是实施法律法规的重要主体，必须带头尊法、学法、守法、用法。要深入贯彻党的十八届四中全会精神，坚持运用法治思维和法治方式深化改革、推动发展、化解矛盾。坚持“法定职责必须为、法无授权不可为”，严格依照法定权限和程序行使权力、履行职责。制定政府议事规则和重大行政决策程序规定，确保制度科学、程序正当、过程公开、责任明确。抓好跨部门、跨行业综合执法试点，提高规范文明执法水平。大力推进政务公开，全面公开政府预决算、部门预决算和“三公”经费预决算，加强政府系统门户网站建设，完善政府信息发布制度。强化监督制约，自觉接受人大及其常委会的法律监督、工作监督和政协的民主监督，主动接受司法监督、舆论监督和社会监督，强化监察、审计监督和政府内部层级监督，确保政府权力在阳光下运行。

（五）加强廉洁政府建设，反腐倡廉要有新成效。为政清廉才能取信于民。要把守纪律讲规矩摆在更加重要的位置，坚持“一岗双责”，严格落实党风廉政建设责任制，加强惩治和预防腐败体系建设。持之以恒地落实中央“八项规定”，严格控制会议和文件数量，严格执行公务接待、办公用房等规定，完成公务用车制度改革。建立完善统一的公共资源交易平台，切实把权力关进制度的“笼子”。全面推进预算绩效管理，所有公共资金一律接受审计监督，做到“花钱必问效，无效必问责”。切实开展好党风廉政建设巡查工作，有腐必反、有贪必肃，坚决查处和严惩各种腐败行为，树立政府为民务实清廉的良好形象。

各位代表，潮平两岸阔，风正一帆悬。让我们更加紧密地团结在以习近平同志为总书记的党中央周围，在省委、省政府和市委的坚强领导下，拼搏进取，扎实苦干，坚决打赢跨越赶超三年攻坚战第三战役，为建设冀鲁豫三省交界科学发展先行区、创造聊城人民更加美好的幸福生活做出新的更大的贡献！
